# Ethical questioning in arts and health-based research: propositions and reflections

**DOI:** 10.3389/fsoc.2023.1249606

**Published:** 2023-11-17

**Authors:** Taiwo Afolabi, Luba Kozak, Calum Smith

**Affiliations:** ^1^Department of Theatre, University of Regina, Regina, SK, Canada; ^2^Centre for Socially Engaged Theatre, University of Regina, Regina, SK, Canada; ^3^University of Johannesburg, Johannesburg, South Africa; ^4^Theatre Emissary International, Lagos, Nigeria; ^5^Department of Interdisciplinary Studies, University of Regina, Regina, SK, Canada; ^6^Population Health, University of Oxford, Oxford, United Kingdom

**Keywords:** ethical questioning, art and health, reflection, social justice, ethics of care

## Abstract

Ethical questioning is a framework for considering the ethical implications and practices in research and is used as a tool for thinking about the connections between art and health. It enables researchers and practitioners to gain a deeper understanding of the emotional dimensions in the field of art and health. In this paper, we propose that ethical questioning, grounded in the principles of ethics of care, can foster a more reflexive and holistic approach to understanding the concept of well-being. We also propose that adopting ethical questioning as a methodology, which requires intentional self-reflection and recognition of positionality, can expose and challenge conventional knowledge hierarchies, resulting in more ethical research outcomes and relationships between researchers and participants. Ultimately, our hypothesis proposes that ethical questioning holds the potential to offer an actionable practice that demonstrates ethics of care.

## Introduction: ethical questioning in art, health, and well-being research

Ethical questioning is “the process of asking practice-oriented and critical questions that have ethical implications in order to better understand or challenge the system one is working in” (Afolabi, 2021a, p. 353). EQ takes on the canons of reflexivity, praxis, and social justice. These values anchor EQ in order to turn ethics into a political act (Afolabi, 2021b). EQ’s framework has three stages: reflection *before-action*, *in-action*, and *on-action* (Afolabi, 2021b). Based on the framework, EQ provokes the act of questioning through a series of questions (Afolabi, 2021b). One of the Feminist founders of ethics of care theory, Virginia Held, posits that ethics of care is both a value and a practice, but ultimately it is premised on a heightened attention to the needs of others that uses an epistemological process that continuously questions what moral choice is best while embraces the influence of emotion instead of rejecting it (Held, 2006, p. 10). Noddings (1982), another founder of ethics of care, argues that a caring relationship is the core of human existence and consciousness (Sander-Staudt, 2023). Ethics of care offers an alternative framework to Kantian deontology or Utilitarianism and is often compared to virtue ethics, but some theorists like Sander-Staudt maintain that ethics of care is unique because it prioritizes care as a way to address injustices (Sander-Staudt, 2006). Rather than considering ethics of care as an abstract concept, EQ offers a tangible and pragmatic entry point “…to better understand or challenge the system one is working in” (Afolabi, 2021b, p. 353). By engaging in intentional reflection ‘before-in-and-on action’ and asking questions from intersectional perspectives (such as environmental, political, and social), EQ fosters a relational and inquiry-based process (Afolabi and Llewellyn, 2022). This intentional approach is in contrast with transactional or result-driven mindsets, encouraging stakeholders to reflect on their assumptions and biases (Hankivsky, 2004). The interconnections between art and health have been of interest to researchers who have studied it using both quantitative and qualitative research methods (Stuckey and Nobel, 2010; Leavy, 2023). Fraser and Al Sayah (2011) pinpoint a particular rise of interest in the relationship between art and Western medicine that can be traced back to the emergence of health-promoting posters after World War II, positioning it as a theme within historical contexts. The arts challenge dominant knowledge creation and dissemination, offering alternate ways of thinking and understanding its impact on health (Capous-Desyllas and Morgaine, 2018, as cited in Leavy, 2009; Barone and Eisner, 2012). Although art can be both therapeutic and empowering, art in health-related contexts requires intentional reflexivity to consider the emotional welfare of others and other ethical implications (Lankston et al., 2010; Boydell et al., 2012). This paper argues that adopting ethical questioning as a framework enables practical engagement with the ethics of care, addressing the need for reflexive practice (Aluwihare-Samaranayake, 2012).

We engage with the ethical questioning (EQ) framework in our different disciplines and spaces; applied theatre, medical humanities, and visual art. Structurally, the article starts with (§1) the authors’ positionality to centre the ways our diverse experiences, exposures and expertise have informed our choices and the development of an inquiry-based process, followed by a reflection on why self-reflection and positionality can play such a key role within this space. Next, (§2) an overview of existing scholarship in arts and health-based research and introduces the idea of how ethics and care and ethical questioning in art-based research can offer new perspectives, elaborating on the role that arts-based approaches (when supported by an ethics of care approach) can play in disrupting knowledge hierarchies. Finally, (§3), through a series of case studies, the authors explore the ways in which ethical questioning guides research on art, health and well-being to consider how ethical questioning and the principles of ethics of care, can (re)shape health-based research and the crucial role that positionality and ethical questioning can play within the field of art and health. The paper concludes with key points of convergence and divergences.

## Positionality as an approach to ethical questioning

Built on reflectivity, EQ takes the practice of positioning seriously. Positionality has the potential for deep reflection, context building and value checks. Starting with oneself rather than the other is essential. Self-positionality, the socio-political, cultural, economic and geographical context, creates one’s identity and the ways one’s identity influences and informs one’s biases, perceptions and outlook on the world. EQ starts with social identity mapping (that is, identifying features that makeup one’s context and worldview) (Jacobson and Nida, 2019). Social identity mapping requires asking self-revealing questions that reveal the position one may hold, the values attached to one’s identity and emotional connections to the details of one’s social identity. The culture and practice of questioning to identify ethical realities is the concern of ethical questioning (Afolabi, 2021b; Afolabi and Llewellyn, 2022).

Lokugamage et al. (2022) argue that art has the opportunity to disrupt ‘historically biased, epistemically rigid, hierarchical thinking’. Art can sit in a liminal space and reveal to us areas that have previously been overlooked. Like ethical questioning, art is able to increase our understanding of, or pose a challenge to, the system one is working in. At this stage, it is worthwhile to reflect on positionality. With the goal of adopting a critical stance or attitude towards our own health-based research practice with the goal of engaging in a process of continuous adaptation and learning, it is valuable to reflect on why art can be perceived to occupy this liminal space.

Public health education, naturally, takes as its starting point lessons and thinking patterns from modern medicine. To examine the positionality of the researcher within this space, it is valuable to examine the cultural and contextual inputs to a medical worldview that prizes objectivity. Daston and Galison (2007) argue that the scientific visual representations which are used (and have historically been used) in medical tuition are indicative of a three-stage process, from ‘truth-to-nature,’ to mechanical objectivity, to trained judgement. In the first, scientists standardised the variability of nature. In the second, there was a belief that nature could be depicted without human intervention, such that research processes and outcomes could be seen as truly objective. In the third, graphs and stylised illustrations are used to capture specific dimensions of certain entities/biomedical occurrences. In the latter, the subjectivity of the scientist is not absent. Rather, the expert’s eye is required in order to decipher the meaning of schematic representations.

Nevertheless, asking practice-oriented and critical questions in order to better understand the values we bring to the table indicates how fully the normative value ascribed to ‘objectivity’ in research is embedded in our worldview. This, at times, can lead to an instrumentalization of art within the sphere of public health; whereby art’s involvement in public health education or messaging is as knowledge translation or awareness raising. This is an artefact of the idea that ‘objectivity’ takes precedence, and the ‘subjective’ nature of art is such that it may be useful secondarily; to translate or make accessible the research results gained through ‘objective’ measures. Combining reflection on the value of art as a way of bridging the ‘hidden third’ with ethical questioning works within the framework of reflecting on an action within the triad cycle of ‘reflection-before-in-and-on action’ (Afolabi and Llewellyn, 2022). More specifically, ethical questioning operates as a framework because it depends on a continual process of reasoning, writing, and conducting ethical research, through self-reflection and awareness, which aims to humanise research by acknowledging limitations (Afolabi, 2021b). It is, therefore, valuable to reflect on the way that art can be used to tackle rigid knowledge hierarchies which can begin to unravel and problematise the epistemo-ethics (Teo, 2019) of research and tuition.

### Taiwo

As a Nigerian who had initial training and exposure to theatre within the context of development, my first experience of health-based research was a bittersweet one. This is because a “developing” country like Nigeria means there are many development projects that come to us from the international communities without necessarily our input. For instance, different international development agencies champion public health-related initiatives on topics such as HIV/AIDs, breastfeeding, and toilet hygiene in the country. This also means the agenda has been preset. Projects are tied to development-related initiatives such as Millennium Development Goals (MDGs), later turned into Sustainable Development Goals (SGDs). I had my own share. I was part of a community theatre project, *Skul Konekt.* This project was already determined by the funding partners, where, the theatre artists that participated in the project were called upon to use the instrument of theatre to create awareness about HIV/AIDS (Afolabi, 2018). Hence, as participants in such projects, we did not have a say in the thematic direction, processes and methods. Although international organisations turned to local experts (mostly scholars and academics), this does not necessarily mean this kind of local expertise has all the knowledge. The political realities of the selection process and the over-centralization of the academy (educational institutions) in such projects continue to have ripple effects on the project design, execution and evaluation.

In addition, in the bid to involve the community (that is the subject of the project), many partners always look for ways to engage the community, hence the use of art and theatre as tools for community engagement and developing communication, among others. The over-instrumentalization of art did not necessarily create the opportunity for researchers, organisations and agencies to consider art an essential part of designing the project itself but rather only as a tool to disseminate the result (or the already made agenda). While the power of storytelling for educating the people (mostly top-down approach) and, in the process, foster participation, art offers affect-driven and sensorial impact that cannot be quantified.

Fast forward to my applied theatre work in a “developed” country like Canada, the principles are still the same even though there are nuances to these issues depending on the community involved, partners and the thematic directions, among others. I engage in art-based methodologies to explore certain social issues, such as care (as in the example below). Ethical questioning and arts-based methods are central to these community explorations.

### Luba

Canadian-born but of proud Ukrainian heritage, I am a scholar who specialises in early modern British art and culture, a combination that makes me keenly aware of how my positionality is influenced by multicultural perspectives. Secondly, I am an animal liberation activist, painfully aware of how my being a homosapien positions me in a role of power and privilege over those with different voices, but also instils a sense of moral responsibility to disrupt hegemonic narratives and care for others. Thirdly, as a researcher, I frequently navigate the tension between the expectations of being an impartial analyst and a knowledge creator guided by my individual experiences and perspectives.

I perceive art and health as complementary and historically interconnected concepts. My interest in health is rooted in my personal experience with the public health system through my father, who underwent a life-saving kidney transplant. He also spent a lengthy stay in the hospital after contracting COVID-19, which caused him to suffer from temporary memory loss. I witnessed how this affected his mental health, and while he cannot accurately recall much during this traumatic time, he can say with certainty that he remembers seeing the artwork on the walls of the hospital corridors. As a result of my father’s lifelong health struggles, I have spent significant time in hospitals and around medical professionals, where a picture of a mountainous landscape or an abstract contemporary artwork has always been in my peripheral vision. Were these images intended for visitors, patients, or the medical staff? Are there more nuances to the story of art in medical spaces? My family history has also inspired me to think about the correlations between art and health, based on my grandfather’s passion for both medicine and music. He worked as a radiologic technologist in the mid-20th century Ukraine, which was then part of the Soviet Union but found a sense of community and an artistic outlet through singing in a professional choir, where many of his doctor colleagues also sang.

Academically, I gained a foundational knowledge of art and health from a university graduate course titled “Mapping Illness,” taught by Dr. Randal Rogers at the University of Regina. The course explored how the field of medicine was understood and represented in art history. From an art historical perspective, I learned that art can serve a dual purpose of documenting and expressing health-related issues where it serves as a material manifestation of individual experiences. However, ethical considerations are necessary when interpreting the representation of medicine in historical artworks and thinking critically about what they can teach us about health in contemporary contexts. Professionally, I collaborated with two doctors by assisting them with thematically curating clinical rooms with artworks on loan from the Regina Hospital Art Organization, which I will talk about subsequently.^1^ This opportunity allowed me to partake in an interdisciplinary partnership where art was enthusiastically embraced into the medical space and allowed me to observe the way the doctors engaged in the creative process of curating and visual story-telling, similar to the way the caregivers are included in the Project Care. Improving the experience of patients was the principal reason behind the curatorial project, yet patients did not have input and the doctors based aesthetic decisions on their own tastes. What would a more collaborative approach in the curation of the rooms, between doctors, patients, artists and/or art professionals look like? What ethical questions were not considered in this case?

### Calum

I grew up in a rural area of the UK and was educated in a state school before studying in Bristol, Cambridge and Oxford. I moved, somewhat unexpectedly, from studying philosophy to studying and working in public health. In studying a course called ‘Death, Dying and Disease’ as an undergraduate I was introduced to the role that art, poetry, and film can have in helping us understand and unpack health and illness. The mutual relationship between art and health has been a key part of my work since.

I was driven to study public health, amongst other things, as a result of an incident in which a family member had a drug overdose. It made me (apologies for the language) adopt a ‘I just want to do shit’ mindset, the view that some stuff is not fair, and my social and health environment (within the UK) is set up in a way that does not really have all the solutions. A clumsy activist’s approach to public health policy analysis. But the more I’ve studied in this space, the more I’ve realised how the spaces where I’ve worked and learned have shaped the way I approach health policy research, and the role that art can play within that. Not just in terms of approach, but in terms of what good research should look like. In a way, that’s probably quite predictable. Within public health, and within academia more generally, ‘rational objectivity’ holds a valuable, if complicated, position. At times, the championing of some sort of ‘rational objectivity’ (with ‘objectivity’ being the place of ‘rationality’ and ‘subjectivity’ being the place of ‘emotion’) can obfuscate the invaluable role that art (and expressions of the ‘personal’ more generally) can have in increasing knowledge. For instance, within public health, as within philosophy and ethics, we are often taught that the best research is the research that exists on a higher plane than the researcher. The idea is that whether it is me, you, or anyone else in the world, ‘objective’ observation and methodological rigour are such that the results will (with any luck) look the same. The research subjects or research subject matter are the focus, completely, and the researcher develops their skills over a number of degrees, courses, application rejections, and so on, in such a way that their own personal ‘bias’ is removed from (a) the observations that lead to the results and (b) the analysis of the results. Even qualitative research at times may hold at its core a valued belief in the transparency of, and ‘non-interference’ by, the researcher.

Furthermore, ‘Objective’ and ‘Subjective’ (though as concepts both highly problematised within the academic sphere) seem to fall on either side of the line – objective research is replicable, informative, and unbiased, unlike subjective research. This implies, of course, that the ideal researcher is one who is able to remove all subjectivity from their research. This positivist perspective has been discussed extensively by many others (Schrag, 1992; Bunniss and Kelly, 2010; Park et al., 2020). The trend towards idealising extraction of traces of the personal forms the backbone of my interests here. The trend may mean that a personal connection to the field is as hampering to the research as a financial conflict of interest. My MPhil thesis contained 30,000 words on the history of drug policy and of family narratives of those campaigning to decriminalise drugs, without once mentioning that my 16-year-old cousin overdosed on MDMA a couple of months before I started the degree. The sense was that my personal conviction and emotional response to this was less reliable or convincing as evidence than, for example, statistics (though of course, they often work in partnership – emotional weight ‘opening the door’ for policy change and statistics showing the need for policy change that can help at a population-level).

How is this all related to arts and health? Arts and health is a burgeoning field, with the potential to problematize this binarity. ‘Art’ seems to fall within the subjective, and research into ‘health’ may fall into ‘objective’. A heart is a heart, though an artwork done ‘with a heart’ varies depending on who the perceiver is. As such, if we go along with this logic, and if we are being fully reductive, ‘art and health’ and ‘art in health’ can fall into difficult categories. Art can help health literacy, engage populations with complex research, and help present the world within which research exists. But this separation of ‘art’ and ‘health’ into distinct categories (which seems very pervasive in the environments I work in) is surely false – and potentially a hangover of the same ‘subjective/objective’ divide. And if that divide is all-pervasive, and is brought into arts and health research, that is potentially worrying.

## Ethics of care and ethical questioning in art-based research: ethical approaches and collaborative methods

There is a growing interest in employing art as a research method, but its adoption in health research is more recent, which leaves room for new approaches (Fraser and Al Sayah, 2011). Although Fancourt (2017) attempts to offer valuable insights into the particular role of arts within health specialties, Vickhoff (2023) argues that the specific role of the arts in health remains undetermined. There is also a preference for quantitative evidence in art-based research in health, which may be driven by the heterogeneity of qualitative methods (Daykin et al., 2016; Daykin, 2019). Broderick (2015) advocates for a shift away from an evidence-based practice model towards a trajectory that emphasises historical and socio-cultural values in the transformative and connective aspects of art engagement in healthcare. Art-based research holds the potential to offer revolutionary insights into human behaviour within the health sciences (Gerber et al., 2020). Yet despite its benefits, qualitative research faces ethical challenges for researchers and participants, such as emotional risks associated with researching personal experiences. However, Aluwihare-Samaranayake (2012) proposes reflexive questioning as a solution for more ethical approaches.

Ethical or reflexive questioning is rooted in ethics of care theory because it encourages introspection about the impact of one’s choices on the needs of others, with the ultimate aim of creating a more equitable framework. Care is a normative orientation and in healthcare specifically, is a set of relational actions that improve or restore well-being (Krause and Boldt, 2018). The notion of relationality in care involves attentiveness, responsiveness, and respect in order to foster ethical relationships (Engster, 2005). Art can be used as a tool for dialogue and building caring relationships. For instance, in Frank’s book, *The Wounded Storyteller* (Frank, 1997), he compiles medical stories that exemplify generosity as an integral part of storytelling, including health-related experiences. This act of generosity through the art of storytelling can spark dialogue between practitioners, researchers and other stakeholders which can build caring relationships. However, these relationships require careful ethical consideration. Learning to see the perspectives and needs of others, rather than gaining a capacity to care, is what matters (Gilligan, 2014). Reflective practice using ethical questioning can help facilitate this process.

Ethics of care extends beyond its original definition as a moral theory, first introduced by Carol Gilligan (Gilligan, 1992/1993; Lawrence and Maitlis, 2012), and has gained interest across disciplines (Hamington and Sander-Staudt, 2011; Urban and Ward, 2020; Mathewson, 2022). Ethics of care can help explain what it actually means to care, or what Pettersen (2011) calls a “conceptualization of care” to address empirical challenges and conflicts of interest. However, the practical implementation of ethics of care lacks direction, which reflectional questioning can assist with. Branch (2000) suggests empathy, compassion, and reciprocity as approaches in care, but overlooks the reflective process involved. Brannelly (2018) outlines the steps needed to integrate ethics of care into participatory research as (1) surfacing marginalised experiences and (2) combining theory with action by embracing the qualities of attentiveness, responsibility, competence, responsiveness, and solidarity that are attributed to caring.

Care in various settings requires a grasp of the intricate nature of relational, social, and temporal dimensions that will allow for care ethics to engage in a transformative practice (Barnes et al., 2015). Engaging in EQ can enable researchers to position themselves within the intricate and multifaceted structure of these dimensions through inward reflection, allowing them to assess the distinctive nature of each situation. In doing so, applying EQ through the values of ethics of care to participatory research methods (case studies 2a and 2b), and in the interpretation of an artwork (case study 2c), demonstrates EQ’s potential to transform social disconnects, as well as ethically reshape research designs and the dynamics between researchers, participants, and community.

An ethics of care framework prioritises the needs of others and influences art-based research designs through reflective contemplations before, during, and after action (Fancourt, 2017). EQ thus goes beyond self-interest to recognize individual experiences and perspectives, while also maintaining connections between people as a result of caring for others; in other words, it values difference but does not operate as a dichotomy. For instance, Vickhoff (2023) suggests that aesthetic empathy connects people through shared mental states, but as Aluwihare-Samaranayake (2012) critiques, this assumption disregards individualised experiences and differing viewpoints by presuming that critical consciousness aligns with similar subjective experience. In another instance, the establishment of the artist-researcher as a strategy in health destabilises power structures and contributes to social activism and epistemological changes (Capous-Desyllas and Morgaine, 2018; Gerber et al., 2020).

Genuine collaboration among diverse researchers, practitioners and the community can bring new perspectives to arts-based methods in healthcare and curatorial practices. Creating collaborative teams comprised of researchers and artists is one approach, although debates exist regarding who should engage in art-based research (Capous-Desyllas and Morgaine, 2018). Daykin et al. (2016) support the idea of co-production through consultation and collaboration between stakeholders in the art and health sectors as an effective strategy for overcoming evaluation challenges and improving participant experiences. Daykin et al. (2016) therefore, argues that co-production repositions the researcher from the role of an expert to a partner, creating a more equitable relationship. Furthermore, including researchers in the creation of artwork or its curation allows for the integration of qualitative research data, as well as ongoing reflection (Archibald and Blines, 2021). An ethics of care framework complements this inclusive approach because it not only values the health and well-being of others but also encourages looking inward to consider the importance of self-care and the existential safety of researchers (Groot et al., 2019). In addition to thinking about the collaborative partnerships between researchers and practitioners, EQ can also place importance on participant input, which is not always the case in traditional art-based research, as Ryu (2018) critiques: “Participants’ perspectives on ethical issues in this co-constructive and collaborative approach are absent.”

## Objectivity, knowledge hierarchies, and art as a ‘bridge’

The integrative nature of the role of art in health, and the ability for art to provide participants with perspectives on key interrelated health and ethical issues, points to the ability of art to disrupt knowledge hierarchies within public health research. Steelman et al. (2019) make strong points for the ability of art to be able to cross what they call the ‘hidden third’ (borrowing from conceptualization by Nicolescu (2010)). They argue that art can bridge ‘objective’ and ‘subjective’ worldviews, aligning the head and the heart. Their work looked at changing engagement practices to enhance sustainability, rather than focusing on improving scientific techniques (through working on long-term socio-ecological change with Indigenous peoples from three inland delta regions in Canada).

Lokugamage et al. (2022) argue that ‘historically biased, epistemically rigid, hierarchical thinking’ has led to environmental collapse and a biomedical paradigm that urgently needs to be rethought due to the damaging impacts of a non-reciprocal relationship with nature. They argue that art, along with qualitative research and storytelling, ought to be put on an equal footing with RCTs. Art can disrupt knowledge hierarchies and, they argue (Verhagen, 2009, quoting Bourriaud, 2009), can usher in a helpful ‘Altermodernism’ in which the artist encourages disorientation through tracing lines in different directions of time and space, with the artist becoming a cultural nomad.

One such ramification is the separation of ‘art’ from ‘medicine’. That singing, dance, or theatre somehow exists in a different medical toolkit from prescriptions and medicines. Steelman et al. (2019) argue that art can bridge the ‘hidden third’ between objectivity and subjectivity. Maybe, in bridging this hidden third, art can also be brought more centrally into tuition about what effective healthcare can look like. This is already done in many places globally, and we are cognizant of the fact that our positionality and education are such that we see a separation between ‘art’ and ‘healthcare’ into separate disciplines in ways that others do not.

Objectivity and subjectivity are key concepts within health research, as well as social research (Letherby et al., 2012). As Daston and Galison (2007) argue, ‘objectivity’ is itself a social artefact; a concept that emerged in the nineteenth-century sciences. The claim that the individual ought to ‘be objective’ is itself a normative claim, and highlights the links between ethics and epistemology, or ‘epistemo-ethics’ (Teo, 2019). Through this linkage, Teo (2019) argues, epistemic values become personal virtues. ‘Objectivity’, then, is itself informed by, championed by, and perpetuated by specific historical contingencies. Its promulgation as an ideal to be aimed for within research methodology is, to use the same language, a cultural artefact.

Increasingly, research has examined how epistemology and knowledge production have historically had a significantly narrow scope as a result of Western/colonial orientation. Darder (2019) explores the extent to which Western political and economic interests distort perceptions of (and lead to the absorption or disruption of) knowledge outside the Western purview. Similarly, within research spaces, Galvez and Muñoz (2020) argue, as academics and students may be in some places conducting their research within educational institutions with colonial histories, the codes of research governing practice are bound by (and inherit) specific, contingent ways of working and producing knowledge. Go (2020) analysed racialized exclusions within the discipline of sociology, and found that through its deep epistemic structures, the subject itself perpetuates epistemic exclusion on grounds linked to an empire. Sociology, Harris (2020) argues, adopts a traditional positivist approach rooted in a world built from a colonial perspective. Undervaluing the role of connections to communities being valuable to scientific data, and the use of qualitative methods within scientific study, that it follows is a direct corollary of a research methodology emphasising the value of objectivity. A dominant colonialist perspective can perpetuate a “spectacle of otherness” felt by minorities in academia, who need to legitimise their voices within this colonial space (Jamjoom, 2020). Jamjoom’s auto-ethnographical article exposes some of the “colonialist frameworks” that are still rooted and embedded in academia (Jamjoom, 2020, p. 261).

Within medicine and medical research, the epistemo-ethics (the link between epistemology and ethics) of Western knowledge production lead to intersectoral, sociohistorical and intergenerational inequities that are emblematic of the discipline’s inheritance of colonial thought (Naidu, 2021). Indeed, as Marya and Patel (2021) argue in ‘Inflamed: Deep Medicine and the Anatomy of Injustice’, “colonial medicine cannot admit to a diagnosis for which colonialism is responsible.” The threads woven into the social artefact of ‘objectivity’ as a research methodology have unseen ramifications. Loss of interconnection, cultural sensitivity, planetary damage and environmental degradation are all linked to a positivist framing of knowledge production that, at times, suffers from a lack of awareness of the essential benefits of the inclusion of a pluriverse of voices. Knowledge production and public health issues are both inherently complex, and both would benefit by adopting a research methodology that lets more than a deified ‘objectivity’ into the arena (Denzin and Lincoln, 2018).

Caught up in the adoption of a positivist research methodology are, potentially, two interrelated issues. First, the separation of ‘mind’ from ‘body’, ‘us’ from ‘them’, and ‘human’ from ‘nature’ (Lokugamage et al., 2022). Second, and much covered in the literature, is the assumption that ‘objectivity’ is a positivist research framing due to the exclusion of emotion, personal bias, and so on. This perspective reinforces the idea that research based on personal experience, recognition of emotion, and reference to positionality, is less ‘rigorous’ or useful within academia, including the medical sciences. The perfect ‘objective’ research, it could be argued, leaves the researcher invisible. This, it follows, has knock-on effects on the inclusion of art as a research methodology. As a discipline, art and art production are inherently emotional. The artist is essentially visible in the work they produce, and (modern questions about AI-art production aside), the individual humanity of the artist is an essential part of the process itself. This, it follows, sits in direct opposition to the invisibility of the ‘objective’ researcher.

Critical works have been conducted by researchers in an attempt to disrupt rigid knowledge hierarchies (Santos, 2015; Smith, 2021). Bilgen and Schöneberg (2020) argue for reflexivity in research processes as a tool to help dismantle embedded power hierarchies. Their analysis of non-reflexive research methodologies is worth quoting in full: “We give birth to a baby created of data. We cut its umbilical cord from its mother, the context, and slowly baptise it with the holy waters of theoretical knowledge and sanctify it in a specific technical and/or disciplinary language.” Martinez-Vargas (2020) also stresses the importance of a pluriverse; of diverse knowledge systems.

In total, it is valuable to recognise the importance of art as inherently valuable, as a contributor to good health and wellbeing, and as an effective gateway to, an example of, and study of the application of an ethics of care approach to public health and medicine. The inherent ability of art-based approaches to health care and public health research to incorporate the personal, and the important self-reflexive work required in order to understand both what we bring to the table, and our role within the system we work within.

## Discussion, reflection and case studies

### Discussion one: ‘project care’

#### Context and content

Project Care focused on senior citizens and was held at one of the senior homes in British Columbia. Project Care (henceforth PC) was a reminiscence theatre that involved creating a performance for senior citizens who had dementia, physical disability and their caregivers. The project was inspired by the idea of institutionalised care and the price placed on care. As an artist-researcher of African descent, I was fascinated and conflicted at the same time with the idea of institutionalised care when I first arrived in Canada. It was interesting to see a congregate living environment designed to meet seniors’ functional, medical, personal, social and housing needs. I was interested in unsettling the discourse around ageing, care and health, and the role of funds in accessing such levels of care. Through the use of music and devised performance, participants (senior citizens, their caregivers and the research group) created space for participation and fun. According to one of the caregivers, the opportunity for senior citizens to participate in storytelling, reminisce about their past experiences etc., was invaluable. The caregivers were fascinated because that was the first-time caregivers were involved in an arts-based project.

#### Reflection

Reflecting-on-action, one of the stages of EQ, Project Care, was an initiative to provide an opportunity for the “developed” society to develop new thinking around institutionalised care. I wanted to offer a new perspective, one that is cultural and different but rooted in the epistemology of my heritage as a person of African descent. For instance, senior citizens shared stories of their families, love and knowledge that would not be passed on to the next generation due to the disconnect between them and their families, but that was no longer possible. Many of them would have loved to share these memories with their loved ones, but that’s not necessarily possible due to the fact that many of them have been “dumped” in care homes. Some of the participants (senior citizens) recanted their memories before they arrived in the home care, and the arts gave them the opportunity to socialise and connect with others in the facility. Apart from the space to create and be nourished, it was an opportunity for them to speak to students, caregivers and community members on pertinent issues. For me, that is one of the ways I use my art to unsettle centres of power and perhaps challenge the status quo by asking critical and thought-provoking questions that will create opportunities for participants (both artists and community members) to think amidst the creative process.

Also, Project Care celebrated seniors and the untiring endeavour of caregivers, which resulted in the creation of stories that served as a way for seniors to share memories. However, it also provided participants with the opportunity to think about caregiving and institutionalised care within the Canadian and North American context. I remember during an interview with one of the caregivers; she was so particular about how institutionalised care has changed family dynamics and familial relationships. Some senior citizens have gained new relationships, while some would have preferred to be with their children or at least grandchildren, especially in this critical period of their lives.

### Discussion two: ‘Co-curating Regina Centre Crossing Family Medicine Unit’

#### Context and content

In the Spring of 2015, I worked as an independent researcher with the Regina Hospital Art Foundation (RHAF), an organisation that depended on public artwork donations that were loaned to medical centres without a designated art department or collection. While working with the RHAF, I was invited to join a curatorial project initiated by two doctors undertaking their residencies at the Regina Centre Crossing Family Medicine Unit (RCCFMU) in Regina. The doctors asked to borrow artworks to display in their clinical rooms, which they hypothesized would positively impact patient emotional experiences. I cannot confirm that the initiative was approved by an ethics board with certainty, but they expressed genuine care for their patients, which made the request compelling. Together, the doctors and I considered the artworks available for loan in the RHAF storage and later met at the RCCFMU clinic to assess the space. The doctors seemed to have a personal appreciation for art and were genuinely excited to be part of the image selection process, so our selection process mainly relied on personal preferences centred on taste, colours, and themes. The doctors had a predetermined vision for how they envisioned the rooms and actively engaged in the decision-making process. Despite my knowledge of formal curatorial practices and standards of aesthetic principles, I realised that the doctors had a strong desire to exert their own influence over the selection and placement of artworks, rather than solely relying on my perceived ‘expert’ opinion. Although I had the qualifications as an art professional to insist on the selection and display of certain artworks, I understood that this project gave the doctors an opportunity to express themselves through art, which was emotionally important for them.

#### Reflection

Reflecting on my experience as part of an interdisciplinary team curating clinical rooms for the Regina Centre Crossing Family Medicine Unit, I recognize the potential EQ could have had on improving the curatorial process and fostering a more inclusive environment. Although the initiative was motivated by the element of care, an ethics of care framework was not formally applied, which would have enabled for deeper reflection and action. In this discussion, I critically examine how EQ could have influenced the project, including aspects like the decision-making process for selecting artworks and the negotiation of their display. I argue that applying EQ as a method could have had a significant impact on the collaborative process while shedding light on the need for patient involvement, which would have influenced the research design and curatorial process as well. As a result, patients would have been empowered as co-curators, ensuring diverse viewpoints and sensitivity to their needs. Ultimately, I believe that EQ would have fostered an equitable and respectful collaborative process, creating a truly inclusive environment for all stakeholders.

Magner (2005) argues that contemporary debates about medicine prioritise healthcare costs and access rather than the art and science of medicine. Although Magner (2005) investigates the idea of ‘art’ through more scientifically thematic and methodological advancements in medicine rather than visual representations of medicine, she asserts that the notions of art and health are closely connected. Indeed, a recognition of the link between art and health was the motivating force behind the curatorial project at the Regina Centre Crossing Family Medicine Unit (RCCFMU) in 2015 with the goal of enhancing patient experiences. The interconnection between arts and health sectors, as Davies et al. (2016) write, involves the placement of art forms (such as paintings) into a setting with the aim of enhancing the health environment. Although most larger hospitals have dedicated art departments and display protocols, I will focus on my experience as an independent researcher/curator for the RCCFMU, reflecting on how EQ could have influenced the collaborative partnership, research design, and outcomes. Afolabi writes, “Ethical questioning is an art in itself that must be learnt. It requires recognising one’s power and privileges–its strengths and limits” (Afolabi, 2021a, p. 354). Furthermore, Hajar (2018) argues that art helps medical students sharpen their observational skills and be more empathetic, which makes them better communicators. Therefore, not only was the curatorial process an emotionally positive experience for the doctors, but it was also beneficial to their training.

Held (2006) posits that ethics of care go beyond the mere idea of what it means to care because it recognizes the value of emotions and relational capabilities that allow morally concerned individuals to understand what is best in interpersonal contexts. Reflecting on this realisation through the lens of ethics of care, I am aware of how caring for the needs of the doctors’ emotional well-being above my own desire to professionally engage in the curatorial process required an ethical line of questioning on my part. After all, Persohn (2021) writes, “The word curator comes from the Latin root curare, meaning ‘to take care of’.”

A relational approach to ethics of care involves other virtues that have a stabilising effect on relationships, such as forgiveness and trust (Maoi, 2018). While the curatorial process could have been more inclusive of my professional input, I opted to prioritise relationship-building and the common objective of benefiting patients. To achieve this, I placed my trust in the doctors’ enthusiasm for art and allowed them to experiment with its implementation by thinking about their needs over my own, believing it would lead to a positive outcome. However, had the doctors embraced EQ as part of their methodology for this project, it would have led to a more equitable and inclusive experience. Daykin et al. (2016) stress that a collaborative approach between stakeholders in art and health sectors shifts the role of the researcher from an expert to a respectful partner, fostering a collaborative space where minds can meet and ideas can be exchanged. Thus, this case underscores the practical application of an ethics of care framework through the utilisation of EQ.

Building on the idea of collaborative approaches in art-based research, Daykin et al. (2016) argue in favour of co-production that can enhance participant experiences. An important aspect that was overlooked in the RCCFMU curatorial project was a lack of patient input and collaboration. For instance, art in hospitals is generally well-received by patients and staff, but certain artworks impact individual psychological responses (Cusack et al., 2010; Lankston et al., 2010). Although the doctors and I had the best of intentions to care about the emotional health of patients, these individuals were not consulted. Using an ethics of care framework to ethically question the *doing* of ethics (Afolabi, 2021a) in this curatorial project would have highlighted how necessary it was to include the perspectives of all stakeholders. An ethics of care approach would also explain, as Held (2006) argues, the reason why we are obligated to care for others, which is based on feelings of empathy for unfamiliar people through sympathetic relatability. And for the doctors, using an EQ framework would have added a practical dimension to their obligation to care for others with whom it was their moral duty to build professional relationships with to best serve them as medical practitioners.

Fancourt (2017) makes a strong case for the importance of collaborating with community members in arts and health research in order to develop partnerships and secure funding. For instance, in their article “Take a walk in someone else’s shoes,” Gillibrand et al. (2023) draw on co-produced participatory arts-based projects as a way to demonstrate the benefits of participatory arts for health research development. One of the examples they provide is based on a series of audio podcasts called “Hidden” that brought together community members and both art and science professionals as a way to enhance the art of storytelling on the topic of COVID-19 vaccination experiences. While the authors of this article do not explicitly engage with ethics of care theory, the researchers’ reflective approach in this project aligns with a form of EQ, which could have been enriched by integrating an ethics of care framework. As Aluwihare-Samaranayake (2012) suggests, a critical consciousness lens could bridge the gap between researchers and participants through reflective thinking and transparency.

### Discussion three: Rembrandt’s *The Anatomy Lesson of Dr Nicolaes Tulp*

#### Context and content

Death and life; teachers and pupils; academicians and criminals; Rembrandt Van Rijn’s *The Anatomy Lesson of Dr Nicolaes Tulp* (Rijn, 1632) is filled with visual juxtapositions that the technique of chiaroscuro further emphasises. The renowned surgeon Dr. Nicolaes Tulp (1593–1674) commissioned Rembrandt van Rijn (1606–1669) to paint the group portrait *The Anatomy Lesson of Dr Nicolaes Tulp* (Ploeg and Buvelot, 2005). The painting portrays the physician and praelector Dr. Tulp teaching an anatomy lesson to a group of doctors who were members of the Amsterdam Guild of Surgeons (IJpma IJpma et al., 2006). In the artwork, Dr. Tulp demonstrates the intricacies of the human body by lifting a tendon on the forearm of a cadaver with the use of forceps. The cadaver is positioned on a table at the centre of the composition, sharing the focal point with the image of Dr. Tulp. Anatomy demonstrations were performed as a popular public event in the seventeenth century and in this case, the cadaver has been identified as Adriaen Adriaensz, also known as Aris Kindt, who was a recently hanged criminal convicted of robbery (Ploeg and Buvelot, 2005). Drawing on Rembrandt’s *The Anatomy Lesson of Dr Nicolaes Tulp* as a case study, I illustrate how EQ, guided by principles of ethics of care, can influence interpretations of Western health and medicine in art.

#### Reflection

I have selected Rembrandt’s *The Anatomy Lesson of Dr Nicolaes Tulp* as a case study because it provides unique insights on health and social relations within medicine that are relevant to those issues with which ethics of care is concerned and impact ethical modes of questioning by researchers, practitioners, and viewers. The painting can be understood to depict broader social issues, including hierarchies, class disparities, and perceptions of self-image, as well as serve as a model of medical pedagogy. Applying EQ, guided by ethics of care, to the reading of such art historical works can unveil the transformative potential of art to offer empowering narratives for marginalised figures by drawing on the concepts of empathy and compassion as it relates to social justice. As I will demonstrate, *The Anatomy Lesson* teaches us about the importance of justice and the need for compassionate care towards others through an acknowledgement of social power dynamics.

Upon learning that the body of the cadaver in the painting is that of the criminal, Aris Kindt, we as the contemporary spectator, are confronted with an ethical dilemma: is it justified to (mis)use his body for medical purposes without his consent due to his lower social status? Held (2006) argues that a caring person does not care for the sake of it being a virtuous quality, but because they are moral subjects and take responsibility. Additionally, Slote (2007) links empathy, or “empathetic care ethics,” with social justice and caring for the unfamiliar or impoverished. EQ compels us to consider Kindt’s potential circumstances and reasons, such as poverty, that could have driven him to commit the crime (Di Matteo et al., 2016). Guided by the principles of ethics of care, this kind of EQ encourages us to express more empathy towards others, challenging our perception of Kindt not solely as a criminal, but recognising his ostracised status within his community. Carrabine (2012) asserts that the ethics of representation revolves around interpersonal connections and an acknowledgement of difference. In this transformative moment of EQ, we as spectators adopt a compassionate and sympathetic response to the figure of the cadaver, whose marginalised status becomes ‘surfaced’ (Brannelly, 2018). The recognition of Kindt’s identity as more than a criminal or anonymous cadaver can be understood as a mode of restorative justice by ethically reestablishing his existence in history. This approach also establishes an imagined relationship that crosses time boundaries, made possible through an ethics of care framework and its connection to social justice (Held, 2006).

*The Anatomy Lesson* also emphasises the tensions between the concepts of spectacle and education. On the one hand, the painting depicts an anatomy lesson being taught, which implies a positive learning opportunity. On the other hand, zooming in on the particular part of the painting that portrays the dissection of the cadaver’s body can be understood as a gruesome spectacle of entertainment for both the viewers outside of the canvas as well as for the other figures in the painting, judging by their different facial expressions and gestures. The ethical implications of balancing the educational value with moral considerations in the painting therefore present us, the spectators, with another ethical dilemma about the spectacle of visually representing violence and suffering, despite the educational intent. Carrabine (2012) refers to this concept as the ‘power of images’, which highlights the importance of sociological understanding of the relationship between aesthetics, ethics, and justice present.

Rembrandt’s *The Anatomy Lesson* depicts power structures, made evident by Dr. Tulp’s stoic central pose, suggestive of his authoritative and distinguished status that dictates our understanding of medical education based on power dynamics between educators and students. On the topic of teaching and learning methods relating to operating theatres, Lyon (2004) suggests that the attitudes and demeanour of surgeons and students affect teaching and learning outcomes. EQ draws attention to the social structures of power and privilege (Afolabi, 2021a), and by reading the painting through this lens, we can consider how Dr. Tulp’s position influences teaching and learning experiences. Are discriminatory attitudes towards others, especially those from different social classes, a consequence of social hierarchies within the medical field? Do pupils have the ability to incorporate their own techniques or different approaches to patient/doctor relationships (Colgan, 2010), or do they model their behaviour and mimic what/how they are taught, without striving for improvement? These lines of questioning can prompt a re-evaluation of learning environments and offer a different kind of teaching model that acknowledges power relations.

The painting can also be understood in terms of moral suffering, defined as being witness to or directly engaging in situations that result in negative moral outcomes (Rushton, 2018). Assessing the moral message connected to the spectacle of suffering in *The Anatomy Lesson* involves EQ, which is informed by compassion and empathy within an ethics of care framework. Are the exposed muscles on Kindt observed with selfish curiosity or as a site where the spectator can experience emotions like compassion and empathy in order to establish a relationship? EQ is centred on respect, a key aspect of ethics of care, which allows us to explore these themes in contemporary contexts (Engster, 2005).

Thinking about representations of health, well-being, and medicine in art historical contexts has significant contemporary relevance and can contribute valuable insights. I see an interconnection between art and health, where historical art can be reinterpreted to offer fresh, empathetic perspectives that reshape contemporary perceptions of health and morality within a broader social context. For instance, we may ask why Rembrandt’s masterpiece, *The Anatomy Lesson*, continues to resonate with modern viewers, despite the fact that we have socially embraced patient confidentiality and a shift away from public displays of dissection like the one exhibited in the painting. Additionally, the way individuals who donate their bodies to science are honoured and glorified further complicates our relationship with the figure in the painting based on the criminal status of the cadaver who did not necessarily consent to being publicly dissected. Does viewing the painting through an ethics of care framework call us to action by highlighting the different dynamics of relationality, therefore reflecting a desire to connect with others in medical contexts, whether it be with practitioners as teachers or cadavers as life-saving donors? Put into practice, since ethics of care prioritises the needs of others, it can help guide us in traversing difficult ethical questions that bring to the forefront the importance of considering social dynamics, building relationships, and implementing policies that are motivated by compassion and emotion, as opposed to simply pursuing biased interests or scientific curiosity (Hankivsky, 2014).

## Conclusion

The paper focuses on some critical issues of ethics and health-based research. From authors’ positionality to variegated thematic exploration which emanated from our experiences, we centre reflexivity, challenge some dogma and offer ways to promote wellness and care in our practice. To promote comprehensive wellness beyond hospital settings, Stuckey and Nobel (2010) suggest community leaders collaborate with researchers to create better healthcare agendas. Care involves imagination and humility (Bourgault and Pulcini, 2018). Embracing collaborative approaches fosters humility through reflective practice, challenging power hierarchies between researchers, practitioners, and the community. Co-production and collaboration in art and health initiatives emphasise the importance of arts in social and emotional well-being by fostering a sense of belonging, improving communication, and encouraging respect through the goal of positive shared experiences (Davies et al., 2016). Finally, art promotes co-habitational thinking and caring, highlighting interconnectedness that encourages a collective and compassionate approach to address existential risks, including those affecting health and well-being. Adopting a reflective approach, a critical framework for ethical questioning, guided by the principles of ethics of care, can foster a fair and respectful collaborative process among stakeholders. This approach has the potential to enhance research design methods and yield more inclusive and comprehensive data results.

## Data availability statement

The original contributions presented in the study are included in the article/supplementary material, further inquiries can be directed to the corresponding authors.

## Author contributions

All authors listed have made a substantial, direct, and intellectual contribution to the work and approved it for publication.

## Funding

The author(s) declare that no financial support was received for the research, authorship, and/or publication of this article.

## Footnotes

^1^[Luba] Regrettably, due to the substantial time that has passed since the project, the names of the doctors involved and their respective contact information have slipped from my memory.

## References

[ref1] Afolabi T. (2018). From ‘*their stigma*’ to ‘*my stigma*’: an examination of the ‘*Skul Konekt*’ project among adolescents in the north-central region of Nigeria. Sage Open (Special Collection–Reproductive Health in Sub-Sahara Africa) 8, 215824401879480–215824401879411. doi: 10.1177/2158244018794800

[ref2] Afolabi T. (2021a). From writing ethics to doing ethics: ethical questioning of a practitioner. Res Drama Educ 26, 352–357. doi: 10.1080/13569783.2021.1880317

[ref3] Afolabi T. (2021b). Theorizing ethical questioning in applied theatre research. Appl. Theat. Res. 9, 99–116. doi: 10.1386/atr_00051_1

[ref4] Afolabi T. Llewellyn J. (2022). Ethics and research-based theatre: reflections from two practitioners. Drama Res. 13, 1–24.

[ref5] Aluwihare-Samaranayake D. (2012). Ethics in qualitative research: a view of the participants’ and researchers’ world from a critical standpoint. Int. J. Qual. Methods 11, 64–81. doi: 10.1177/160940691201100208

[ref6] Archibald M. Blines J. (2021). Metaphors in the making: illuminating the process of arts-based health research through a case exemplar linking arts-based, qualitative and quantitative research data. Int. J. Qualit. Methods 20:160940692098795. doi: 10.1177/1609406920987954

[ref7] Barnes Marian Brannelly T. Ward Lizzie Ward Nicki (Eds). (2015) Ethics of care: Critical advances in international perspective. Bristol: Policy Press

[ref8] Barone T. Eisner E. W. (2012) Arts based research. Los Angeles, CA SAGE

[ref9] Bilgen N. Schöneberg J. (2020). Why positionalities matter: reflections on power, hierarchy, and knowledges in “development” research. Canad. J. Develop. Stud. 42, 519–536.

[ref10] Bourgault S. Pulcini E. (Eds). (2018) Emotions and care: interdisciplinary perspectives, 6. Leuven: Peeters Publishers

[ref11] Bourriaud N. (2009) Altermodern: tate triennial 2009 London Tate Publishing

[ref12] Boydell K. Volpe T. Cox S. Katz A. Dow R. Brunger F.. (2012). Ethical challenges in arts-based health research. Int. J. Creat. Arts Interdiscipl. Pract. 11, 1–17.

[ref13] Branch J. (2000). The ethics of caring and medical education. Acad. Med. 75, 127–132. doi: 10.1097/00001888-200002000-00006 10693842

[ref14] Brannelly T. (2018). An ethics of care research manifesto. Int. J. Care Caring 2, 367–378. doi: 10.1332/239788218X15351944886756

[ref15] Broderick S. (2015) (Mis)interpreting arts and health: What (Else) can an arts practice do?. Doctoral thesis. Dublin Dublin Institute of Technology

[ref16] Bunniss S. Kelly D. R. (2010). Research paradigms in medical education research. Med. Educ. 44, 358–366. doi: 10.1111/j.1365-2923.2009.03611.x 20444071

[ref17] Capous-Desyllas M. Morgaine K. (Eds). (2018) Creating social change through creativity anti-oppressive arts-based research methodologies. 1st. Cham: Springer International Publishing

[ref18] Carrabine E. (2012). Just images: aesthetics, ethics and visual criminology. Br. J. Criminol. 52, 463–489. doi: 10.1093/bjc/azr089

[ref19] Colgan R. (2010) Advice to the young physician on the art of medicine. 1st. New York: Springer US.

[ref20] Cusack P. Lankston L. Isles C. (2010). Impact of visual art in patient waiting rooms: survey of patients attending a transplant clinic in Dumfries. JRSM Short Reports 1, 1–55. doi: 10.1258/shorts.2010.010077 21234115 PMC2994357

[ref21] Darder A. (2019) Decolonizing interpretive research: a subaltern methodology for social change. Oxford: Routledge

[ref22] Daston L. Galison P. (2007) Objectivity. Cambridge: Zone Books.

[ref23] Davies C. Pescud M. Anwar-McHenry J. Wright P. (2016). Arts, public health and the National Arts and health framework: a lexicon for health professionals. Aust. N. Z. J. Public Health 40, 304–306. doi: 10.1111/1753-6405.12545, PMID: 27372460

[ref24] Daykin N. (2019) Arts, health and well-being: a critical perspective on research, policy and practice. 1st. London: Routledge

[ref25] Daykin N. Willis J. McCree M. Gray K. (2016). Creative and credible evaluation for arts, health and wellbeing: opportunities and challenges of coproduction. Arts Health 9, 123–138. doi: 10.1080/17533015.2016.1206948

[ref26] Denzin N. Lincoln Y. (2018) The SAGE handbook of qualitative research. Thousand Oaks: Sage.

[ref27] Di Matteo B. Tarabella V. Filardo G. Tomba P. Viganò A. Marcacci M. (2016). Nicolaes Tulp: the overshadowed subject in the anatomy lesson of Dr. Nicolaes Tulp. Clin. Orthop. Relat. Res. 474, 625–629. doi: 10.1007/s11999-015-4686-y, PMID: 26758442 PMC4746154

[ref28] Engster D. (2005). Rethinking care theory: the practice of caring and the obligation to care. Hypatia 20, 50–74. doi: 10.1111/j.1527-2001.2005.tb00486.x

[ref29] Fancourt D. (2017) Arts in health: Designing and researching interventions. Oxford: Oxford University Press.

[ref30] Frank A. (1997) The wounded storyteller: Body, illness, and ethics. 2nd. Chicago: University of Chicago Press

[ref31] Fraser D. Al Sayah F. (2011). Arts-based methods in health research: a systematic review of the literature. Arts Health 3, 110–145. doi: 10.1080/17533015.2011.561357

[ref32] Galvez E. Muñoz S. (2020). (Re)imagining anti-colonial notions of ethics in research and practice. J. College Stud. Develop. 61, 781–796. doi: 10.1353/csd.2020.0075

[ref33] Gerber N. Biffi E. Biondo J. Gemignani M. (2020). Arts-based research in the social and health sciences: pushing for change with an interdisciplinary global arts-based research initiative. Forum Qual. Soc. Res. 21, 1–15. doi: 10.17169/fqs-21.2.3496

[ref34] Gillibrand S. Hine P. Conyers R. Gravestock J. Walsh C. McAvoy A.. (2023). Take a walk in someone else’s shoes: the role of participatory arts for health research development and training. Res. Involv. Engag. 9:40. doi: 10.1186/s40900-023-00441-6, PMID: 37291659 PMC10249567

[ref35] Gilligan C. (1992/1993) In a different voice: psychological theory and women’s development. Cambridge: Harvard University Press

[ref36] Gilligan C. (2014). The ethic of care. Víctor Grífols Foundat. Bioeth. 30, 1–54. doi: 10.2307/j.ctvjk2wr9

[ref37] Go J. (2020). Race, empire, and epistemic exclusion: or the structures of sociological thought. Sociol. Theory 38, 79–100. doi: 10.1177/0735275120926213

[ref38] Groot B. Vink M. Haveman A. Huberts M. Schout G. Abma T. A. (2019). Ethics of care in participatory health research: mutual responsibility in collaboration with co-researchers. Educat. Act. Res. 27, 286–302. doi: 10.1080/09650792.2018.1450771

[ref39] Hajar R. (2018). What has art to do with medicine? Heart Views 19, 34–35. doi: 10.4103/HEARTVIEWS.HEARTVIEWS_6_18, PMID: 29876031 PMC5965014

[ref40] Hamington M. Sander-Staudt M. (Eds.). (2011) Applying care ethics to business. 1st. Dordrecht: Springer Netherlands.

[ref41] Hankivsky O. (2004) Social policy and the ethic of care. Vancouver: UBC Press.

[ref42] Hankivsky O. (2014). Rethinking care ethics: on the promise and potential of an intersectional analysis. Am. Polit. Sci. Rev. 108, 252–264. doi: 10.1017/S0003055414000094

[ref43] Harris J. (2020). Black on black: the vilification of ‘me-search’, tenure, and the economic position of black sociologists. J. Econ. Race, and Policy 4, 77–90. doi: 10.1007/s41996-020-00066-x

[ref44] Held V. (2006) The ethics of care: personal, political, and global. Oxford: Oxford University Press

[ref45] IJpma F. F. A. van de Graaf R. C. Nicolai J. P. A. Meek M. F. (2006). The anatomy lesson of Dr. Nicolaes Tulp by Rembrandt (1632): a comparison of the painting with a dissected left forearm of a Dutch male cadaver. J. Am. Soc. Surg. Hand 31, 882–891. doi: 10.1016/j.jhsa.2006.02.014, PMID: 16843145

[ref46] Jacobson D. Nida M. (2019). Social identity mapping: a reflexivity tool for practicing explicit positionality in critical qualitative research. Int. J. Qualit. Methods 18:160940691987007. doi: 10.1177/1609406919870075

[ref47] Jamjoom L. (2020). A spectacle of otherness: an autoethnography of a conference presentation. Law Soc. Inquiry 16, 261–277. doi: 10.1108/QROM-12-2018-1708

[ref48] Krause F. Boldt J. (Eds.). (2018) Care in healthcare reflections on theory and practice. 1st Basingstoke Palgrave Macmillan 31314223

[ref49] Lankston L. Cusack P. Fremantle C. Isles C. (2010). Visual art in hospitals: case studies and review of the evidence. J. R. Soc. Med. 103, 490–499. doi: 10.1258/jrsm.2010.100256, PMID: 21127332 PMC2996524

[ref50] Lawrence T. Maitlis S. (2012). Care and possibility: enacting an ethic of care through narrative practice. Acad. Manag. Rev. 37, 641–663. doi: 10.5465/amr.2010.0466

[ref51] Leavy Patricia. (2009) Method meets art: Arts-based research practice. New York Guilford Press

[ref52] Leavy Patricia. (2023) Research design. 2nd. New York: The Guilford Press.

[ref53] Letherby G. Scott J. Williams M. (2012) Objectivity and subjectivity in social research. California: SAGE Publications

[ref54] Lokugamage A. Chetwynd C. Harris M. (2022). Biomedical research and global sustainability: throwing off the straightjacket of hierarchical thinking, making space for nomadic thinking. Decol. Subvers. 3, 1–10.

[ref55] Lyon P. (2004). A model of teaching and learning in the operating theatre. Med. Educ. 38, 1278–1287. doi: 10.1111/j.1365-2929.2004.02020.x, PMID: 15566539

[ref56] Magner L. (2005) A history of medicine. 2nd. Boca Raton: Taylor & Francis.

[ref57] Maoi G. (2018). “Fundamentals of an ethics of care” in Care in healthcare reflections on theory and practice. eds. Boldt J.. (Cham: Palgrave Macmillan) 31314223

[ref58] Martinez-Vargas C. (2020). Decolonising higher education research: from a university to a pluri-versity of approaches. South Afr. J. Hig. Educat. 34, 112–28. doi: 10.20853/34-2-3530

[ref59] Marya R. Patel R. (2021) Inflamed: deep medicine and the anatomy of injustice. UK: Allen Lane.

[ref60] Mathewson J. (2022) Ethical journalism: Adopting the ethics of care. Abingdon, Oxon: Routledge

[ref61] Naidu T. (2021). Modern medicine is a colonial artifact: introducing decoloniality to medical education research. Acad. Med. 96, S9–S12. doi: 10.1097/ACM.0000000000004339, PMID: 34380933

[ref62] Nicolescu B. (2010). Methodology of transdisciplinarity. Levels of reality, methodology of the included middle and the complexity. Transdiscip. J. Eng. Sci. 1, 18–37. doi: 10.22545/2010/0009

[ref63] Noddings N. (1982) Caring: a feminine approach to ethics and moral education. Berkeley: University of CA Press.

[ref64] Park Y. Konge L. Artino A. R. (2020). The positivism paradigm to research. Acad. Med. 95, 690–694. doi: 10.1097/ACM.0000000000003093, PMID: 31789841

[ref65] Persohn L. (2021). Curation as methodology. Qualit. Res. QR 21, 20–41. doi: 10.1177/1468794120922144

[ref66] Pettersen T. (2011). The ethics of care: normative structures and empirical implications. Health Care Anal. 19, 51–64. doi: 10.1007/s10728-010-0163-7, PMID: 21207152 PMC3037474

[ref67] Ploeg P. Buvelot Q. (2005) Royal Picture Gallery Mauritshuis: a princely collection. The Hague: Mauritshuis.

[ref68] Rijn R. (1632). *The anatomy lesson of Dr Nicolaes Tulp*. Oil on canvas, 169.5 cm × 216.5 cm. At: Mauritshuis, The Hague. no. 146.

[ref69] Rushton C. (2018) Moral resilience: transforming moral suffering in healthcare. New York: Oxford Academic

[ref70] Ryu H. (2018). Participants' perspectives on ethical issues in arts-based health research: a qualitative study (Order No. 10680373). ProQuest Dissertations & Theses Global.

[ref71] Sander-Staudt M. (2006). The unhappy marriage of care ethics and virtue ethics. Hypatia 21, 21–39. doi: 10.1111/j.1527-2001.2006.tb01126.x

[ref72] Sander-Staudt M. (2023) Ethics of care. Internet of encyclopedia of philosophy. Available at: https://iep.utm.edu/care-ethics/

[ref73] Santos B. (2015) Epistemologies of the south: justice against epistemicide. London: Routledge

[ref74] Schrag F. (1992). In defense of positivist research paradigms. Educ. Res. 21, 5–8. doi: 10.2307/1176838

[ref75] Slote M. (2007) The ethics of care and empathy. London: Routledge

[ref76] Smith L. (2021). Decolonizing methodologies: research and indigenous peoples. 3rd. London: Zed

[ref77] Steelman T. Andrews E. Baines S. Bharadwaj L. Bjornson E. R. Bradford L.. (2019). Identifying transformational space for transdisciplinarity: using art to access the hidden third. Sustain. Sci. 14, 771–790. doi: 10.1007/s11625-018-0644-4, PMID: 31149316 PMC6503894

[ref78] Stuckey H. L. Nobel J. (2010). The connection between art, healing, and public health: a review of current literature. Am. J. Public Health (1971) 100, 254–263. doi: 10.2105/AJPH.2008.156497, PMID: 20019311 PMC2804629

[ref79] Teo T. (2019). “Academic subjectivity, idols, and the vicissitudes of virtues in science: epistemic modesty versus epistemic grandiosity” in Psychological studies of science and technology. eds. O’Doherty K. Osbeck L. Schraube E. Yen J. (London: Palgrave Macmillan), 31–48.

[ref80] Urban P. Ward L. (eds.) 2020) in Care ethics, democratic citizenship and the state. eds. Petr. 1st ed (Cham: Springer Nature).

[ref81] Verhagen M. (2009). The nomad and the altermodern: the tate triennial. Third Text 23, 803–812. doi: 10.1080/09528820903371206

[ref82] Vickhoff B. (2023). Why art? The role of arts in arts and health. Front. Psychol. 14:765019. doi: 10.3389/fpsyg.2023.765019, PMID: 37034911 PMC10075207

